# Myofibroblast fate plasticity in tissue repair and fibrosis: Deactivation, apoptosis, senescence and reprogramming

**DOI:** 10.1111/wrr.12952

**Published:** 2021-06-19

**Authors:** Wolfgang Merkt, Yan Zhou, Hongwei Han, David Lagares

**Affiliations:** 1 Fibrosis Research Center, Center for Immunology and Inflammatory Diseases, Division of Pulmonary and Critical Care Medicine, Massachusetts General Hospital, Harvard Medical School, Boston, Massachusetts; 2 Department of Hematology, Oncology and Rheumatology, Internal Medicine V, University Hospital of Heidelberg, Heidelberg, Germany; 3 Department of Physiology, Xiangya Medical School, Central South University, Changsha, China

**Keywords:** apoptosis, cellular senescence, fate, fibrosis, myofibroblast, plasticity, reprogramming, tissue repair

## Abstract

In response to tissue injury, fibroblasts differentiate into professional repair cells called myofibroblasts, which orchestrate many aspects of the normal tissue repair programme including synthesis, deposition and contraction of extracellular matrix proteins, leading to wound closure. Successful tissue repair responses involve termination of myofibroblast activities in order to prevent pathologic fibrotic scarring. Here, we discuss the cellular and molecular mechanisms limiting myofibroblast activities during physiological tissue repair, including myofibroblast deactivation, apoptosis, reprogramming and immune clearance of senescent myofibroblasts. In addition, we summarize pathological mechanisms leading to myofibroblast persistence and survival, a hallmark of fibrotic diseases. Finally, we discuss emerging anti-fibrotic therapies aimed at targeting myofibroblast fate such as senolytics, gene therapy, cellular immunotherapy and CAR-T cells.

## 1 | INTRODUCTION: MYOFIBROBLAST FATES UPON COMPLETION OF TISSUE REPAIR

**FIGURE 1:**
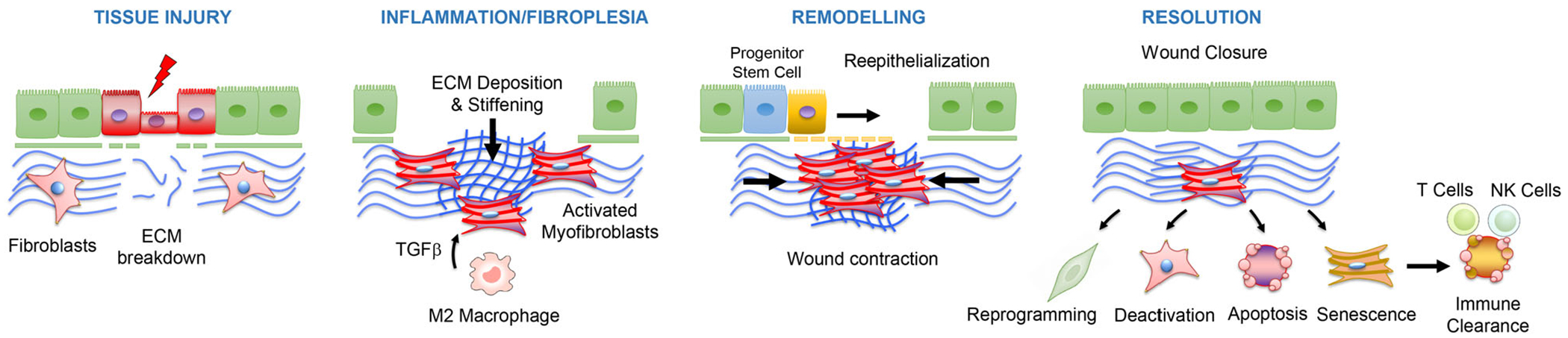
Myofibroblast fate upon termination of the tissue repair programme. Following tissue injury, extracellular matrix (ECM) breakdown and inflammatory cytokines such as TGFb promote fibroblast differentiation into activated myofibroblasts, which are characterized by increased synthesis of ECM and the neo-expression of alpha smooth muscle actin (α-SMA), a protein that confers these cells a hyper-contractile phenotype. These two cellular activities are essential for promoting wound closure and reepithelialization. Successful tissue repair responses involve termination of myofibroblast activities via alternative mechanisms including deactivation, apoptosis, immune clearance of senescent myofibroblasts by T and natural killer (NK) cells and reprograming

During homeostasis, scattered tissue-resident fibroblasts actively maintain the fitness of connective tissues through processes of synthesis, degradation and remodelling of extracellular matrix (ECM) proteins. These processes are controlled through a series of feedback mechanisms involving both mechanical and biochemical cues that instruct fibroblast to produce or degrade the ECM. Upon tissue injury, ECM breakdown, haemostasis and inflammation unleash fibroblast pro-repair responses during normal wound healing, ultimately promoting fibroblast proliferation and differentiation into professional repair cells named myofibroblasts.^1^ Activated myofibroblasts are characterized by increased synthesis of ECM proteins including type I collagen and the neo-expression of alpha smooth muscle actin (α-SMA), a protein that confers these cells a hyper-contractile phenotype.^2^ These two cellular activities are essential at promoting wound closure and preventing blood loss and infections.^3^ During the late phase of tissue repair, myofibroblasts orchestrate a series of cellular responses aimed at fully regenerating or partially restoring parenchyma function.^1^ Successful tissue repair responses involve termination of myofibroblast activities in order to prevent maladaptive repair responses characterized by pathologic fibrotic scarring.^4,5^ Given the importance of myofibroblasts in balancing regeneration and fibrosis, a growing body of research is currently addressing the mechanisms regulating myofibroblast activities during the resolution phase of wound repair and fibrosis.^6^ In this review, we will summarize the state of the art regarding cellular and molecular mechanisms limiting myofibroblast activities during normal tissue repair, including myofibroblast deactivation, apoptosis, reprogramming and immune clearance of senescent myofibroblasts (Figure 1). We will also discuss escape mechanisms leading to myofibroblast persistence and the development of fibrosis. Finally, we will discuss emerging therapeutic strategies aimed at reducing myofibroblast activities to treat fibrotic diseases.

## 2 | MYOFIBROBLAST APOPTOSIS DURING WOUND HEALING

Successful tissue repair requires timely resolution of the inflammatory and fibroproliferative responses, ultimately leading to reduction in tissue cellularity. Early reports published in the 1990s proposed that myofibroblast apoptosis is a major mechanism responsible for myofibroblast clearance in skin repair, as evidenced by morphological changes and accumulation of DNA double-strand brakes in α-SMA+ cells.^4,5,7^ More recent studies have validated this concept in animal models of tissue injury, further supporting the notion that programmed cell death plays a central role in wound repair by promoting controlled elimination of myofibroblasts during the resolution of the healing programme.^7–10^ Whereas the mechanisms that promote fibroblast proliferation and activation into myofibroblasts have been extensively studied and reviewed,^11–13^ the molecular underpinnings that control myofibroblast apoptosis remain poorly understood. We have recently shown that differentiation of fibroblasts into activated myofibroblasts associates with augmented predisposition to apoptosis in these cells,^6,14,15^ increasing the readiness of myofibroblasts to activate programmed cell death. Thus, activated myofibroblasts are “primed for death,” a mechanism that enables rapid elimination of these cells upon completion of the tissue repair programme. On the molecular level, this process is controlled by dynamic changes in the “mitochondrial apoptotic priming” of myofibroblasts across the different phases of the tissue repair programme.

### 2.1 | Mitochondrial priming controls myofibroblast predisposition to apoptosis

Apoptotic priming is dynamically regulated by a molecular rheostat involving the BCL-2 family of proteins, which compromises both pro-apoptotic and anti-apoptotic members that control the mitochondrial or intrinsic pathway of apoptosis.^14,16^ This pathway is triggered by intracellular pro-apoptotic stimuli such as oxidative stress, DNA damage or oncogene activation, ultimately inducing mitochondrial outer membrane permeabilization (MOMP), cytochrome C release and activation of executioner caspases. Strong inducers of mitochondrial apoptosis such as the BCL-2 family member BIM are highly expressed in activated myofibroblasts compared to resting fibroblasts.^6,17^ The mechanisms increasing levels of BIM and other pro-apoptotic molecules in myofibroblasts are poorly understood, although increased production of mitochondrial reactive oxygen species (ROS) has been associated with mitochondrial damage and apoptotic signalling in these cells.^15,18,19^ Platelet-derived growth factor (PDGF) signalling has been similarly shown to prime cancer-associated myofibroblasts for apoptosis.^20^ Despite being tonically primed for death, myofibroblasts evade apoptosis by upregulating pro-survival molecules that directly inhibit pro-death signalling. We and others have shown that pro-apoptotic activities of BIM are selectively blocked by the anti-apoptotic BCL-2 family protein BCL-X_L_, which directly binds to and sequesters BIM in myofibroblasts’ mitochondria.^17^ Up-regulation of pro-survival BCL-2 proteins including BCL-X_L_ is induced by growth factors, cytokines and ECM cues. For instance, the cytokine transforming growth factor beta (TGFβ), the vasoactive peptide endothelin-1 (ET-1) and PDGF have been shown to promote an apoptosis-resistant phenotype in myofibroblasts by activating pro-survival signalling pathways such as FAK-PI3K-AKT and ABL.^21–27^ Similarly, matrix cues including ECM stiffness promote myofibroblast evasion of apoptosis by activating survival mechanotransduction pathways such as integrin-FAK-ROCK-YAP/TAZ signalling.^6,12^ We and others have recently shown that mechanical activation of YAP/TAZ directly regulates expression of the anti-apoptotic protein BCL-X_L_ in primed for death myofibroblasts, ensuring their survival.^17^ In addition, TGFβ also blocks the extrinsic pathway of apoptosis by inhibiting Fas–Fas ligand (FasL) signalling, a mechanism dependent on blockade of ceramide generation by sphingomyelinase.^28^ Taken together, increased mitochondrial priming in activated myofibroblasts predisposes these cells towards apoptosis. Primed for death myofibroblasts are highly dependent on the activation of survival pathways and expression of anti-apoptotic proteins of the BCL-2 family members. In the following section, we discuss cellular and molecular mechanisms that selectively inhibit these pro-survival mechanisms during the resolution phase of wound healing, ultimately leading to myofibroblast clearance via apoptosis.

### 2.2 | Mechanisms inducing apoptosis in “primed for death” myofibroblasts

#### 2.2.1 | Matrix softening and degradation

Upon injury, the wound is filled with a provisional matrix composed of fibrin, fibronectin and collagens, which matures into scar tissue during the remodelling phase of the healing programme. As the tissue repair programme progresses, the mechanical properties of the granulation tissue change, in particular by increasing its stiffness and resistance to deformation. These changes in matrix stiffness have been shown to directly modulate the biology of fibroblasts during wound repair by promoting their mechano-activation into myofibroblasts.^2,12^ Previous reports have shown that dermal stiffness increases from 1 kPa in healthy skin tissues up to 20 kPa in 7 days post-wounding and 50 kPa in mature granulation tissue 14 days post-injury.^29^ At this point, wound closure is almost completed due to full activation of hyper-contractile myofibroblasts.^30^ Of note, massive degradation of the ECM is observed once the wound is closed, which associates with myofibroblast apoptosis.^31^ Several reports have shown that the degree of myofibroblasts apoptosis appears to correlate with severity of ECM stiffness and tension drop. For instance, 15% of activated myofibroblasts undergo apoptosis following release of anchored collagen gels in vitro from high to low tension,^31^ whereas 40% of activated myofibroblasts activate apoptosis when tension drops from very high to zero.^32,33^ In vivo, releasing the edges of a previously splinted excisional wound, which leads to tension drop from high to medium, causes apoptosis in 10% of the cells, albeit the percentage of apoptotic myofibroblasts was not calculated.^34^ Mechanistically, ECM degradation and softening have been shown to promote myofibroblast apoptosis by inhibiting mechanically activated FAK signalling and reducing levels of pro-survival BCL-X_L_ in primed for the myofibroblasts in vitro and in vivo models.^31,34^ More recently, destiffening of the ECM has been shown to activate the pro-apoptotic Mdm4-p53 pathway and promote myofibroblast apoptosis.^35^ The cellular mechanisms promoting ECM degradation and softening remain poorly understood but may involve reprogramming of injury-associated macrophages that switch their phenotype from pro-repair to matrix-degrading phenotype during the resolution of tissue repair.^36–38^ Accordingly, molecules involved in the degradation and uptake of collagen such as uPARP and Mfge8 have shown to promote ECM degradation and prevent tissue fibrosis in mouse models.^39,40^

#### 2.2.2 | Growth factor withdrawal

During tissue repair, primed for death myofibroblasts become addicted to cytokines and growth factors that activate pro-survival signalling pathways, ensuring their survival. Levels of TGFβ, ET-1 and PDGF, which are released by inflammatory cells and platelets during the inflammatory phase of the healing programme, are reduced during the resolution phase,^6,41,42^ putting forward the hypothesis that withdrawal of survival signals upon completion of the tissue repair programme leads to myofibroblast apoptosis.^27,43^ While this hypothesis has not been demonstrated in vivo, several reports have shown that starvation of cultured myofibroblasts associates with reduced FAK-PI3K-AKT signalling and apoptosis. Together, growth factor “dependence” may control myofibroblast clearance during wound healing.

#### 2.2.3 | Pro-apoptotic factors

Another plausible mechanism involves factors that specifically inhibit survival signalling in myofibroblasts. For instance, fibroblast growth factor 1 (FGF1), interleukin-1β (IL-1β) and hepatic growth factor (HGF) have been shown to deactivate the FAK-PI3K-AKT pathway and induce apoptosis of myofibroblasts from skin granulation tissue, but not in healthy control fibroblasts. Similarly, the prostaglandin PGE2 blocks AKT signalling and increases the sensitivity of myofibroblasts to Fas-induced apoptosis via the extrinsic pathway. In this regard, activation of the extrinsic pathway of apoptosis by FasL and TNF-related apoptosis-inducing ligand (TRAIL) has been similarly reported to promote myofibroblast apoptosis in vivo.^8,44,45^ Accordingly, loss of pro-apoptotic Fas signalling in myofibroblasts impairs homeostatic tissue repair resolution and promotes pulmonary fibrosis in mice.^8^ In humans, bronchoalveolar lavage fluid from patients in the resolution phase of lung repair, but not from patients with active lung injury, induces myofibroblast apoptosis, further suggesting that myofibroblasts are actively and timely eliminated via apoptosis.^46^

Together, the termination of myofibroblasts’ pro-repair activities emerges as a tightly regulated process in which both intrinsic apoptotic priming as well as spatiotemporal regulation of mechanical and biochemical factors fine-tune the activation of myofibroblast apoptosis during the resolution phase of the repair programme.

#### 2.2.4 | Myofibroblast senescence and immune clearance

In addition to self-clearance by apoptosis, acquisition of a senescent phenotype has been shown to reduce myofibroblast activities and promote their clearance by cytotoxic immune cells. The role of cellular senescence in normal tissue repair remains poorly understood, although it appears to be a mechanism to arrest myofibroblasts and to limit their pro-repair activities^47,48^ during the resolution phase of the tissue repair programme in the liver,^49^ lungs,^50,51^ skin,^52,53^ kidney,^54^ oral submucosa^55^ and pancreas.^56^ Mechanistically, the matricellular protein CCN1 has been shown to promote myofibroblast senescence in vivo in the context of liver and skin injury, promoting liver regeneration and limiting fibrosis in mouse models.^52,57^ In addition, the senescent phenotype appears to target myofibroblast for recognition and clearance by immune cells via not yet fully understood mechanisms. A growing body of evidence has reported that the senescence-associated secretory phenotype (SASP) of senescent myofibroblasts includes pro-inflammatory chemokines and cytokines that lead to the recruitment and activation of cytotoxic immune cells. Thus, senescent myofibroblasts appear to be programmed to be eliminated by the immune system. This concept has been demonstrated in multiple studies confirming the role of immune cells in eliminating senescent myofibroblasts during tissue repair, and that impaired immune clearance of senescent cells leads to overwhelming fibrosis.^49,58–60^ Several immune cell types have shown the capacity to eliminate senescent cells in vitro and in vivo, including innate immune cells such as natural killer (NK) cells, macrophages and neutrophils,^61^ but also T cells.^59,62,63^ In the context of wound healing, NK cells expressing TRAIL have been involved in the resolution of the tissue repair programme by promoting apoptosis of senescent myofibroblasts and other cell types.^44,49^ Similarly, cytotoxic CD4+ T cells expressing pro-apoptotic FasL have been shown to eliminate myofibroblasts in the context of lung injury.^45,64^ In addition, senescent dermal myofibroblasts can be cleared out by CD8+ T cells and NK cells ex vivo.^65^ Mechanistically, the acquisition of a senescent phenotype leads to up-regulation of stress ligands in myofibroblasts and other cell types, which can be recognized by specific receptors expressed in cytotoxic lymphocytes.^66^ Stress signals upregulated in senescent myofibroblasts, but not healthy cells, selectively target them for apoptosis by immune cells.^67–69^ For instance, up-regulation of MIC-A/B and ULBPs in senescent myofibroblasts have been shown to activate both NK cells and a subset of CD8+ T cells via the NKG2D receptor.^44,49,65,70^ NKG2D activation leads to NK and CD8+ T activation and the release of cytotoxic granules containing perforin and granzymes that induce myofibroblast apoptosis. Accordingly, both NKG2D- and perforin-deficient mice show accumulation of senescent liver myofibroblasts and increased fibrosis in mouse models of liver injury.^64,70^ More recent reports have shown that senescent myofibroblasts may escape immune clearance in pathological settings by co-expressing inhibitory ligands or decoy receptors that block immune cell function. For instance, upregulation of the decoy death receptor Dcr2^64^ or the inhibitory molecule HLA-E^65^ in senescent myofibroblasts have been associated with resistance to immune cell killing. In addition, leukocytes have been involved in myofibroblast apoptosis by secreting interferon gamma (IFNγ), which facilitates myofibroblast apoptosis by NK cells in the context of liver injury.^71–73^ Finally, myofibroblast apoptotic bodies are subsequently phagocytosed by macrophages via efferocytosis, which associates with anti-inflammatory and wound healing resolution responses.^74,75^

## 3 | MYOFIBROBLAST DEACTIVATION AND REPROGRAMMING DURING WOUND HEALING

Genetic lineage-tracing studies in mice have recently demonstrated that myofibroblasts undergo dynamic phenotypic transitions through different activation states in vivo.^10,76–78^ These studies show that myofibroblasts get deactivated upon termination of the normal tissue repair in vivo, a phenotype characterized by reduction in α-SMA levels, contractile activities and ECM synthesis.^79,80^ The mechanisms promoting myofibroblast deactivation remain poorly defined, although several reports have demonstrated a major role for peroxisome proliferator-activated receptor gamma (PPARγ) at promoting myofibroblast deactivation by blocking the TGF-β pathway. In the liver, ~50% of total myofibroblasts get deactivated during the resolution of murine liver fibrosis via up-regulation of PPARγ, albeit these reprogrammed cells acquire a phenotype distinct from quiescent hepatic stellate cells.^10,76^ In the lungs, lineage-tracing studies show that myofibroblasts get similarly deactivated by turning into lipofibroblast-like cells during lung fibrosis resolution, a process associated with PPARγ up-regulation.^81^ In the heart, periostin-traced myofibroblasts get deactivated to resting fibroblasts upon injury resolution in myocardial infarction models, a mechanism associated with re-gaining expression of the transcription factor Tcf21.^77^ Accordingly, overexpression of Tcf21 deactivates hepatic myofibroblasts in vitro and protects mice from CCl4-induced liver fibrosis and diet-induced steatohepatitis.^82^ In the skin, lineage-tracing studies have also demonstrated that dermal myofibroblasts get deactivated via reprogramming to adipocytes through activation of the BMP-ZFP423 pathway upon termination of the wound healing programme.^78^ Further studies have shown that the transcription factor MyoD acts as a critical switch controlling myofibroblasts activation and deactivation in vivo by modulating the TGF-β pathway.^83^ Together, these studies elegantly demonstrate the capacity of wound myofibroblasts to get deactivated in vivo via reprogramming into resting cells or converting to another cell type. The molecular mediators controlling these processes remain poorly understood, although soluble factors such as PGE2, BMP4, PDGFB, HRAS, EGR4 and FGF-1 have been shown to block the TGF-β pathway in cultured myofibroblasts.^80,84,85^ In addition, matrix softening has been also reported to induce myofibroblast deactivation. Several studies have shown that stiffness-activated myofibroblasts get deactivated when plated on soft matrices.^86–89^ Similarly, desplinting and releasing the wound edge has been shown to induce myofibroblast deactivation in vivo.^29,90^ In addition to these cell-intrinsic mechanisms, bystander cells including immune cells and adipocytes have been shown to promote myofibroblast deactivation. For instance, myofibroblasts in aortic valve stenosis get deactivated after transcatheter aortic valve replacement and such deactivation is triggered by IL-1β and TNF-α released from M1 macrophages in post-surgery serum.^91^ Similarly, adipocyte-conditioned medium significantly decreases the expression of α-SMA and ECM components in skin myofibroblasts through BMP4 mediated activation of PPARγ signalling.^85^ More recent studies have shown that activated myofibroblast can get directly reprogrammed into adipocytes via BMP signalling during wound healing, contributing to tissue repair and regeneration.^78^

## 4 | MYOFIBROBLAST PERSISTENCE LEADS TO TISSUE FIBROSIS

**FIGURE 2:**
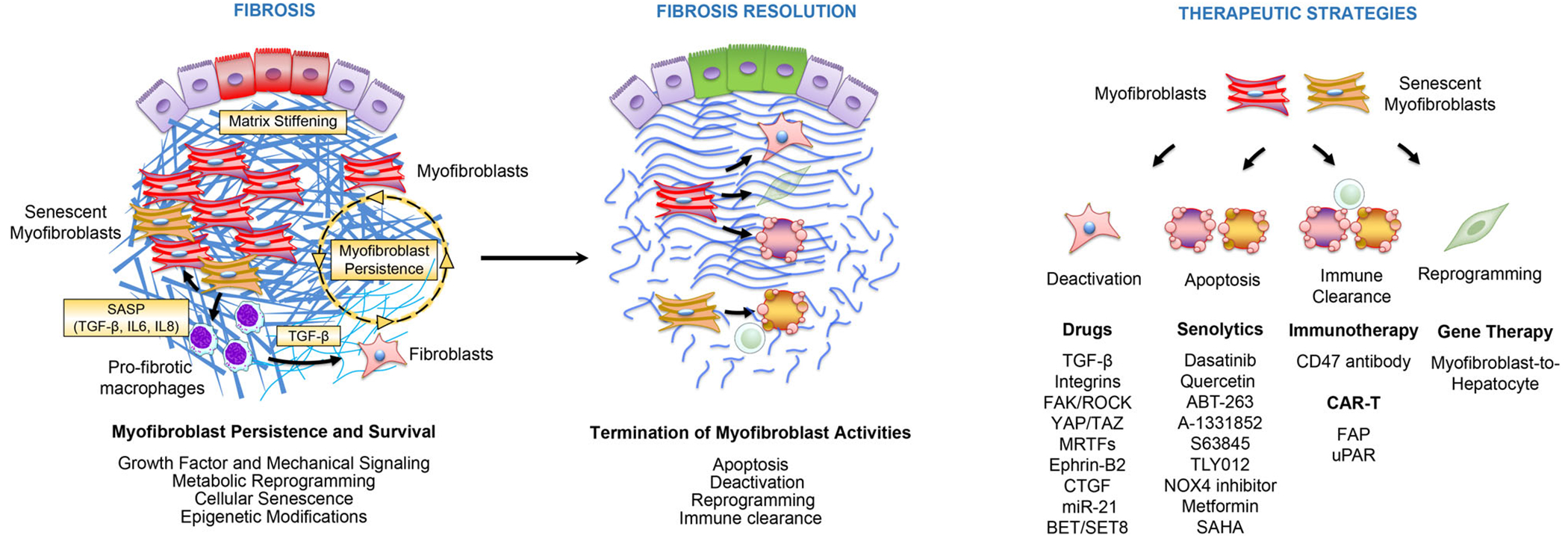
Targeting myofibroblast fate for the treatment of established fibrosis. Myofibroblast persistence is a hallmark of fibrotic diseases characterized by progressive tissue scarring. The molecular mechanisms that promote myofibroblast persistence include pathological growth factor signalling (e.g. TGF-β pathway), mechanical signalling induced by matrix stiffness, cellular senescence and the senescence-associated secretory phenotype (SASP: TGF-β, IL6, IL8) as well as metabolic and epigenetic reprogramming. This knowledge has paved the way for new therapeutic strategies aiming at reversing fibrosis by targeting myofibroblast fate and promoting myofibroblast apoptosis and immune clearance, deactivation and reprogramming. These novel treatments include senolytics, gene therapy, cellular immunotherapy and CAR-T cells, and may not only reverse fibrosis but also regenerate fibrotic tissues

Human fibrotic diseases including idiopathic pulmonary fibrosis (IPF), systemic sclerosis (SSc, scleroderma), liver cirrhosis, cardiac fibrosis and chronic kidney disease (CKD) are associated with high morbidity and mortality.^92^ Persistent myofibroblast activation is a hallmark of these fibrotic diseases and associates with progressive tissue scarring and organ failure.^6,41^ The molecular pathways that promote myofibroblast persistence beyond the resolution phase of tissue repair remain poorly understood, although recent studies have identified mechanisms that keep myofibroblasts in an activated state by promoting evasion of apoptosis, cellular senescence as well as metabolic and epigenetic reprogramming (Figure 2).

### 4.1 | Evasion of apoptosis: Addiction to TGF-β1 and mechanical signalling

Persistent growth factor signalling has been shown to promote myofibroblast evasion of apoptosis by activating pro-survival pathways. The pro-fibrotic cytokine TGF-β promotes an apoptosis-resistant phenotype in myofibroblasts by activating the pro-survival FAK-PI3K-AKT and p38 MAPK pathways.^21,25,93^ In addition, TGF-β blocks mitochondrial apoptosis by modulating levels of the BCL-2 family of proteins. For instance, TGF-β inhibits the pro-apoptotic sensitizer protein BAD, upregulates pro-survival proteins such as BCL-2 and BCL-X_L_ and decreases BAX to BCL-2 ratio via cellular Abelson (c-Abl) signalling.^27,94–97^ In addition to blocking the intrinsic apoptotic pathway, TGF-β has been also shown to inhibit FasL-mediated activation of the extrinsic apoptotic pathways in scleroderma myofibroblasts by blocking ceramide production.^94,98^ Restoration of Fas signalling upon treatment with PGE2, a lipid mediator which is found deficient in IPF, increases Fas receptor expression and sensitizes myofibroblasts to apoptosis by inhibiting AKT signalling.^99,100^

In addition to TGF-β signalling, increased matrix stiffness in fibrotic conditions has been shown to block apoptotic signalling in myofibroblasts via activation of integrin-mediated mechanotransduction pathways.^6,17^ In fibrotic diseases, myofibroblasts promote progressive matrix stiffening due to excessive deposition and crosslinking of ECM proteins, locking themselves in a mechanical positive feedback loop. This vicious cycle amplifies fibrotic responses by enhancing myofibroblast activation and persistence.^101^ We and others have shown that stiff matrices directly activate FAK, a pro-survival mechanosensitive protein activated by integrin signalling.^22,102^ FAK mediates Rho/Rho-associated protein kinase (ROCK) activation and increases acto-myosin contractility, which leads to activation of transcriptional coactivators yes-associated protein (YAP)/transcriptional coactivator with PDZ-binding motif (TAZ) and myocardin-related transcription factor-A (MRTF-A)/MRTF-B, widely recognized as major mechanotransducers.^103,104^ We and others have shown that stiffness-activated YAP/TAZ promotes up-regulation of BCL-X_L_ in myofibroblasts and that FAK–YAP–TAZ–BCL–X_L_ pathway is hyperactivated in dermal myofibroblasts from patients with scleroderma.^15,105^ Similarly, the mechanosensitive pathway FAK–ROCK–MRTF–BCL-2 promotes survival and persistence of IPF fibroblasts.^106^ In addition, mechanical signalling inhibits the extrinsic apoptotic pathways by blocking acid sphingomyelinase and reducing Fas expression in myofibroblasts.^28,94,98^

### 4.2 | Metabolic reprogramming

Myofibroblasts undergo metabolic reprogramming in order to sustain the high-energy demand associated with their ECM hyper-synthetic and contractile phenotype. A growing body of evidence suggests that metabolic reprogramming, characterized by increased aerobic glycolysis, mitochondrial oxygen consumption and ROS production, promotes myofibroblast persistence and survival.^19,107–112^ This notion that myofibroblast bioenergetics are linked to their persistent activation state has been demonstrated by studies showing that inhibition of glycolysis in myofibroblasts leads to rapid down-regulation of α-SMA.^19,113^ In addition, metabolic reprogramming and NOX4-mediated ROS production in myofibroblasts have been associated with mitochondrial dysfunction characterized by fragmented mitochondria and impaired activation of mitochondrial apoptosis in these cells.^19,107,111^ Accordingly, pharmacological activation of the energy-sensor with metformin promotes mitochondrial biogenesis, restores myofibroblast sensitivity to apoptosis and reverses established lung fibrosis in mouse models.^18^ Moreover, metabolic reprogramming associates with enhanced glutamine metabolism in IPF-derived fibroblasts, leading to increased expression of the apoptosis inhibitors XIAP and surviving.^114^ More recently, increased glutaminolysis and glycolysis have been linked to Ca2+-dependent metabolic reprogramming and myofibroblast persistence via epigenetic mechanisms.^115^

### 4.3 | Epigenetic mechanisms

Epigenetic modifications including DNA methylation, histone modification and regulation by non-coding RNA such as microRNAs (miRs), have been recently associated with myofibroblast activation and persistence.^111,116^ Difference in DNA methylation has been described in healthy control fibroblasts compared to fibroblasts isolated from patients with IPF, CKD and scleroderma.^117–122^ Mechanistically, TGF-β signalling induces expression of DNA methyltransferase 3A (DNMT3A) and DNMT1 in myofibroblasts, leading to increased DNA methylation and modulation of pro-fibrotic gene expression.^123^ In this regard, DNA hypermethylation silences endogenous anti-fibrotic genes such as PPARγ, a mechanism controlled by methyl cap binding protein 2 (MeCP2), a transcriptional repressor that binds to methylated DNA.^124^ Accordingly, MeCP2-deficient mice showed protection from fibrosis in the lungs and liver in mouse models.^124^ Similarly, hypermethylation of anti-fibrotic genes such as THY-1 and FLI1 leads to down-regulation of these molecules and increased type I collagen synthesis in fibrotic fibroblasts isolated from patients with IPF and scleroderma.^118,125^ Moreover, hypermethylation and subsequent silencing of the proapoptotic protein P14^ARF^ promotes an apoptosisresistant phenotype in IPF fibroblasts.^126^ Noteworthy, selective methylation of three CpG islands in ACTA2 gene (the encoding gene of α-SMA) has been also shown to reduce α-SMA expression in myofibroblasts.^127^ On the contrary, DNA hypomethylation and consequent transcription up-regulation of pro-fibrotic genes including COLIVA1, TGFBR3, SMAD3, SMAD6 has been described in kidney fibrosis.^121^ Acetylation and methylation of histone proteins have been similarly associated with changes in chromatin structure and increased gene transcription in myofibroblasts. For instance, hyperacetylation of histone H4 by P300 has been shown to be required for TGF-β-induced type I collagen expression in myofibroblasts.^128^ Similarly, histone 3 lysine 9 (H3K9) hypermethylation of the *PPARGC1A* gene promoter suppresses expression of PGC1α via the CBX5/G9a pathway and promotes sustained activation of IPF fibroblasts.^129^ Moreover, decreased histone acetylation and increased histone 3 lysine 9 trimethylation (H3K9Me3) of the Fas promoter reduces Fas expression and promotes resistance to Fas-mediated apoptosis.^130^ Accordingly, pharmacological inhibition of histone deacetylases with suberoylanilide hydroxamic acid (SAHA) reverses established lung fibrosis by inducing myofibroblast apoptosis via BCL-X_L_ down-regulation.^131^ On the contrary, histone deacetylation by the histone deacetylase 4 (HDAC4) has been reported to suppress the activated myofibroblast phenotype by inhibiting α-SMA expression.^132^ miRs suppress gene expression via direct binding to 3′ UTR of target. In myofibroblasts, down-regulation of miR-29a and miR-133a has been associated with myofibroblast persistence and survival, and that increase in miR-29a promotes myofibroblast apoptosis.^133^ On the contrary, up-regulation of miR-21 has been shown to promote myofibroblast survival by reducing levels of pro-apoptotic BAX.^134^ Moreover, up-regulation of miR-22 has been shown to promote myofibroblast persistence and survival by driving cellular senescence.^135^

### 4.4 | Cellular senescence

Myofibroblast senescence has been traditionally associated with the resolution of the normal tissue repair programme and that elimination of senescent myofibroblasts prevents the development of fibrosis.^52,57^ However, a growing body of evidence suggests that impaired clearance of senescent myofibroblasts shifts their pro-repair activities to pro-fibrotic via persistent secretion of pro-inflammatory and pro-fibrotic mediators^50,136–141^ . In this regard, the SASP of pro-fibrotic senescent myofibroblast has been shown to contain pro-inflammatory cytokines (IL-1α, IL-1β, IL-6 and IL-8), pro-fibrotic cytokines (TGF-β, PDGF) and ECM proteins (fibronectin, collagens).^50,142^ Human fibrotic fibroblasts derived from patients with various fibrotic diseases display a senescent phenotype.^143–146^ Noteworthy, senescent myofibroblasts activate pro-survival mechanisms including up-regulation of BCL-X_L_, which promotes their persistence within fibrotic tissues. In humans, senescent myofibroblasts from patients with IPF show elevation of p16 and p21.^50,51,55,136^ Mechanistically, acquisition of a senescent phenotype appears to be regulated by the DNA damage response,^55^ independently from TGF-β signalling.

## 5 | TARGETING MYOFIBROBLAST PERSISTENCE FOR ANTI-FIBROTIC THERAPY

Organ fibrosis has been traditionally thought to be irreversible. However, fibrosis has been shown to regress in humans in response to pharmacological treatment of the underlying pro-fibrotic process. For instance, liver fibrosis can regress after treatment of viral hepatitis, pancreas transplantation can ameliorate diabetic nephropathy, hypotensive drugs can treat hypertensive cardiac fibrosis, and lung fibrosis can resolve substantially in patients with acute respiratory syndrome. These examples have in common that defined fibrogenic injuries exist, and that haltering of these pro-fibrotic insults associates with fibrosis regression. However, the exact aetiology of most human fibrotic disease remains unknown such as in IPF or SSc. In these fibrotic diseases, it is thought that the establishment of self-amplification pro-fibrotic loops leads to progressive fibrotic responses independently from the initial insult. Up to date, only nintedanib and pirfenidone have been approved for the treatment of IPF and scleroderma-associated interstitial lung disease.^147–149^ Although these drugs slow down the rate of progression of fibrosis, they do not halt or reverse established fibrosis.^150^ The mode of action of these drugs remains poorly understood, but they likely inhibit mechanisms involved in myofibroblast formation and collagen synthesis rather than mechanisms promoting fibrosis resolution. Recent insights into the mechanisms promoting myofibroblast persistence and survival have paved the way for new therapeutic strategies aiming at reversing fibrosis by targeting myofibroblast fate. These novel treatments include senolytics, gene therapy, cellular immunotherapy and CAR-T cells, and may not only reverse fibrosis but also regenerate fibrotic tissues (Figure 2).

### 5.1 | Targeting myofibroblast apoptosis

Substantial evidence has demonstrated that activated (senescent) myofibroblasts in fibrotic tissues are prone to undergo apoptosis due to increased mitochondrial priming, as described above. This knowledge of apoptotic priming has led to the development of novel anti-fibrotic strategies aimed at blocking pro-survival mechanisms activated in senescent myofibroblasts, ultimately inducing activation of programmed cell death.

#### 5.1.1 | Senolytic drugs unleash the intrinsic pathway of apoptosis

The so-called BH3 mimetic drugs are small molecules that selectively antagonize anti-apoptotic BCL-2 proteins (BCL-2, BCL-X_L_, BCL-W, MCL-1 and A1) by directly binding to specific shallow hydrophobic grooves.^14^ This knowledge has led to the discovery and development of ABT-737 and ABT-263 (navitoclax, which blocks BCL-2, BCL-X_L_, BCL-W), ABT-199 (venetoclax, a BCL-2 selective inhibitor) and several BCL-X_L_ and MCL-1 mono-selective inhibitors.^14,151–154^ We have recently shown that ABT-263 reverses established dermal fibrosis in a mouse model of scleroderma by targeting myofibroblast for apoptosis.^17^ Mechanistically, ABT-263 directly blocks the pro-survival protein BCL-X_L_, releasing pro-apoptotic proteins such as BIM that ultimately triggers myofibroblast apoptosis. In vitro, ABT-263 induces apoptosis of fibrotic fibroblasts isolated from patients with scleroderma.^17^ In addition, ABT-263 has been recently identified as a potent senolytic agent by targeting senescent cells for apoptosis and reversing lung fibrosis induced by ionizing radiation in mice.^155^ These data validate genetic and pharmacological studies showing that elimination of senescent cells protects mice from lung and kidney fibrosis.^51,156–158^ In the heart, ABT-263 has been shown to eliminate senescent cardiomyocytes and improve cardiac function in mouse models of cardiac ischemia–reperfusion injury^159^ and angiotensin II-induced cardiac hypertrophy and fibrosis.^160^ Importantly, clearance of senescent cells improved myocardial remodelling and diastolic activity along with a higher rate of survival in the event of myocardial infarction.^161^ In the liver, the BCL-X_L_ selective inhibitor A-1331852 treats biliary liver fibrosis in a mouse model of primary sclerosing cholangitis by targeting senescent cholangiocytes and fibroblasts for apoptosis.^162^ More recently, the MCL-1 inhibitor S63845 induces apoptosis of proliferative ductular reactive cells, reducing hepatic fibrosis in a mouse model of cholestasis.^163^

#### 5.1.2 | TRAIL ligands activate the extrinsic pathway of apoptosis

As described above, several mechanisms contribute to reduced sensitivity of myofibroblasts to FasL-induced apoptosis.^164^ In vitro, activation of the cell-surface death receptors (DRs) of the extrinsic pathway of apoptosis has been shown to sensitize myofibroblasts to FasL-induced apoptosis.^165^ Accordingly, selective agonism of DR4 and DR5 with the TRAIL-TLY012 induces myofibroblast apoptosis and reverses established skin fibrosis in a mouse model of scleroderma,^166^ highlighting the therapeutic potential of targeting myofibroblasts for apoptosis via activation of the extrinsic apoptosis pathway. Moreover, PEGylated TRAIL ameliorates liver cirrhosis in rats by inducing apoptosis of activated hepatic stellate cells.^167^

### 5.2 | Cellular immunotherapy

#### 5.2.1 | Harnessing macrophage and T cell anti-fibrotic potential

Harnessing the immune system to treat fibrosis has recently emerged as a promising therapeutic strategy. Recent reports have demonstrated that CD47, a “don’t-eat-me-signal,” is upregulated in scleroderma myofibroblasts and prevents phagocytosis by macrophages.^168^ Accordingly, pharmacological blockade of CD47 leads to myofibroblast clearance by macrophages and reversion of fibrosis in an adaptive transfer model.^169^ Cytotoxic T cells have been similarly involved in myofibroblast clearance by activating the extrinsic pathway of apoptosis. In vivo, myofibroblast persist in FasL-deficient mice subjected to lung fibrosis models, and that reconstitution with FasL^+^ cytotoxic cells promote myofibroblast apoptosis and fibrosis resolution in mice.^45^

#### 5.2.2 | CAR-T cells

CAR-T cells are T-lymphocytes engineered to express a chimeric receptor targeting a specific antigen, thereby triggering T-cell activation and killing of target cells.^170^ Recently, CAR-T cells designed to selectivity eliminate cardiac fibroblasts expressing fibroblast activation protein (FAP) have been shown to reduce cardiac fibrosis and restoration of cardiac function after injury in mice.^171^ Similarly, CAR-T cells selectively targeting senescent cells expressing the urokinase-type plasminogen activator receptor (uPAR) has been shown to treat liver fibrosis in mouse models.^172^

### 5.3 | Targeting myofibroblast deactivation

Over the last 20 years, multiple studies have demonstrated the ability of several agents at treating established fibrosis in preclinical models by promoting myofibroblast deactivation to a low activity state. FDA-approved anti-fibrotic drugs, pirfenidone and nintedanib, have been shown to reduce ECM synthesis and myofibroblast numbers by antagonizing the TGF-β pathway.^173,174^ Accordingly, direct inhibition of TGF-β ligand, TGF-β receptors or integrin-mediated TGF-β activation have been similarly shown to attenuate established fibrosis in mouse models.^175^ Moreover, targeting secondary pro-fibrotic mediators induced by TGF-β such as ephrin-B2, CTGF or ET-1 reduces ECM synthesis in myofibroblasts.^15,176,177^ Similarly, antagonizing TGF-β signalling in fibroblasts with PPARγ agonists reduces matrix deposition both in vitro and in vivo.^175^ In addition, inhibitors of mechanotransduction pathways including FAK, ROCK, YAP/TAZ and MRTFs have been shown to treat established fibrosis by reducing myofibroblast pro-fibrotic activities.^12,178^ Targeting epigenetic modulators such as the histone methylase SET8 with the UNC0379 inhibitor or the bromodomain and extra-terminal (BET) with JQ1 have been shown to deactivate myofibroblasts and treat pre-clinical fibrosis.^179,180^ The list of anti-fibrotic targets aimed at reducing myofibroblast activities is evergrowing and extensible reviewed elsewhere. More recent studies have identified functionally and spatially distinct fibroblast lineages involved in skin repair and fibrosis, supporting the notion that specific pro-fibrotic fibroblasts populations can be targeted for therapeutic treatment.^181–183^ Specifically, the Engrailed-1 fibroblast lineage is endowed with fibrogenic potential and is the primary contributor to ECM synthesis during embryonic development, wounding and fibrosis.^181^ Accordingly, selective targeting of Engrailed-1+ fibroblasts with the CD26 pharmacological inhibitor diprotin A reduced skin scarring in wounding models.^181^

### 5.4 | Targeting myofibroblast reprogramming

Conversion of activated myofibroblasts into epithelial-like cells holds potential for the treatment and regeneration of fibrotic tissues. For instance, fibrogenic injury in the liver leads to loss of hepatocytes and accumulation of activated myofibroblasts. In this regard, recent studies have demonstrated that direct reprogramming of liver myofibroblasts into hepatocytes reverses established liver fibrosis and repopulates the hepatocyte pool in mice. In these studies, lentiviral-mediated ectopic overexpression of the transcription factors FOXA3, GATA4, HNF1A and HNF4A converts mouse myofibroblasts into hepatocyte-like cells and attenuates liver fibrosis.^184^ Similarly, liver myofibroblast reprogramming into hepatocytes using adeno-associated virus (AAV) vectors expressing six hepatic transcription factors including Foxa1, Foxa2, Foxa3, Gata4, Hnf1a or Hnf4a reverses liver fibrosis in vivo.^185^ This novel therapeutic approach may open new avenues for the treatment of chronic liver fibrosis using gene therapy.

## 6 | CONCLUSIONS

Spatiotemporal control of myofibroblast functions is regulated by alternative fates during normal tissue repair and fibrosis. Myofibroblast deactivation, apoptosis, senescence and reprogramming appear to limit pro-repair functions and prevent the development of fibrosis: Whether these alternative myofibroblast fates represent specific or redundant fail-safe mechanisms for limiting myofibroblast functions remains to be investigated. Thus, further research is needed to understand the mechanisms and signalling pathways that determine these specific myofibroblast fates and their functions during normal tissue repair. In this line, recent studies using single-cell RNA sequencing have shown that heterogeneous myofibroblast subpopulations undergo dynamic phenotypic changes during tissue repair and fibrosis, further suggesting that myofibroblast fate plasticity is essential for our body to respond to multiple injury scenarios. Despite these advances, it remains unknown how these different fates are interconnected as well as their temporal and local wiring. A growing body of evidence suggests that persistent myofibroblast activities lead to fibrosis, a manifestation of a pro-fibrotic phenotype associated with impaired or chronic myofibroblast fate. Therapeutic targeting of myofibroblast fates such as induction of apoptosis of (senescent) myofibroblasts, deactivation or reprograming could reverse established fibrosis and potentially regenerate chronically injured tissues. Given that the origin of myofibroblasts depends on specific progenitors present in each tissue (e.g. resident fibroblasts, epithelial and endothelial cells, pericytes and adipocytes), it will be important to understand the contribution and function of specific myofibroblast fates in different organs and systems, which might inform about organ-specific anti-fibrotic strategies. Further research is needed to translate these innovative and promising therapeutic strategies from the bench to the bedside.

## Data Availability

The data that support the concepts described in this manuscript is derived from resources available in the public domain.

## Abbreviations:

α-SMA: α smooth muscle actin
AAV: adenoassociated virus
BET: bromodomain and extra-terminal
c-Abl: cellular Abelson
CKD: chronic kidney disease
DNMT3A: DNA methyltransferase 3A
DRs: death receptors
ECM: Extracellular Matrix
ET: endothelin
FAP: fibroblast activation protein
FasL: Fas ligand
FGF1: fibroblast growth factor 1
H3K9: histone 3 lysine 9
H3K9Me3: histone 3 lysine 9 trimethylation
HDAC4: histone deacetylase 4
HGF: hepatic growth factor
IFNγ: Interferon gamma
IL-1β: interleukin-1β
IPF: idiopathic pulmonary fibrosis
kPa: kilopascal
MeCP2: methyl cap binding protein 2
miRs: microRNAs
MOMP: mitochondrial outer membrane permeabilization
MRTF-A/B: myocardin-related transcription factor-A/B
NK: natural killer
PDGF: Platelet-derived growth factor
PPARγ: peroxisome proliferator-activated receptor gamma
ROS: reactive oxygen species
SAHA: suberoylanilide hydroxamic acid
SASP: senescence-associated secretory phenotype
SSc: systemic sclerosis
TAZ: transcriptional coactivator with PDZ-binding motif
TGFβ: transforming growth factor
TRAIL: TNF-related apoptosis inducing ligand
uPAR: urokinase-type plasminogen activator receptor
YAP: yes-associated protein
